# A Review of Research on Fruit and Vegetable Picking Robots Based on Deep Learning

**DOI:** 10.3390/s25123677

**Published:** 2025-06-12

**Authors:** Yarong Tan, Xin Liu, Jinmeng Zhang, Yigang Wang, Yanxiang Hu

**Affiliations:** Institute of Data Science and Agricultural Economics, Beijing Academy of Agriculture and Forestry Sciences, Beijing 100097, China; tanyarong@baafs.net.cn (Y.T.); zhangjinmeng@baafs.net.cn (J.Z.); wangyigang@baafs.net.cn (Y.W.); huyanxiang@baafs.net.cn (Y.H.)

**Keywords:** deep learning, fruit and vegetable picking robots, goal detection, perception, decision making and control

## Abstract

Fruit and vegetable picking robots are considered an important way to promote agricultural modernization due to their high efficiency, precision, and intelligence. However, most of the existing research has sporadically involved single application areas, such as object detection, classification, and path planning, and has not yet comprehensively sorted out the core applications of deep learning technology in fruit and vegetable picking robots, the current technological bottlenecks faced, and future development directions. This review summarizes the key technologies and applications of deep learning in the visual perception and target recognition, path planning and motion control, and intelligent control of end effectors of fruit and vegetable picking robots. It focuses on the optimization strategies and common problems related to deep learning and explores the challenges and development trends of deep learning in improving the perception accuracy, multi-sensor collaboration, multimodal data fusion, adaptive control, and human–computer interaction of fruit and vegetable picking robots in the future. The aim is to provide theoretical support and practical guidance for the practical application of deep learning technology in fruit and vegetable picking robots.

## 1. Introduction

With the continuous advancement of global agricultural modernization, fruits and vegetables are essential agricultural products whose planting area and production are in-creasing yearly. According to statistics, the global annual output of fruits and vegetables has reached billions of tons, becoming an important part of the agricultural economy [1,2,3]. However, in stark contrast to the increase in production, the harvesting of fruits and vegetables has long relied on manual operation, labor intensity, low efficiency, and rising costs, especially in countries and regions with a shortage of labor resources; the labor cost occupies a significant part of the total cost of fruit and vegetable harvesting. This status quo not only puts pressure on farmers’ economic benefits but also challenges the stability of the supply chain [4,5,6]. In this context, the automation of fruit and vegetable picking has become an inevitable trend in the development of modern agriculture, and picking robots, because of their high efficiency, precision, and intelligence, are considered to be an important way to solve the above problems, which is of great significance in promoting agricultural modernization.

In recent years, with the continuous development of artificial intelligence technology, fruit and vegetable picking robots have ushered in important technological breakthroughs, especially the rapid development of deep learning technology, which has injected new vitality into fruit and vegetable picking robots. In terms of fruit detection, deep neural network structures such as convolutional neural networks (CNNs) have been widely used in fruit and vegetable target recognition and localization tasks, such as the application of the R-CNN family of methods in target detection proposed by Girshick et al. [7], as well as the advantages of YOLO and SSD algorithms in terms of real-time and accuracy [8,9], both of which have been introduced to fruit and vegetable picking scenarios by several research teams. For example, Sa et al. [10] applied Faster R-CNNs to detect apples in real time with a recognition accuracy of over 90%. In addition, deep reinforcement learning (DRL) gradually shows greater potential in path planning and control. By jointly optimizing the sensing results with the robotic arm motion decision, researchers can achieve adaptive adjustment of picking strategies in dynamic environments. For example, Yuqi Liu et al. proposed a deep reinforcement learning strategy combined with expert experience guidance for the path planning of a fruit and vegetable picking robotic arm. The method improves the learning efficiency and performance of the model by introducing expert experience at the early stage of training [11]. Regarding data enhancement and environment simulation, generative adversarial networks (GANs) provide a new means of synthesizing fruit and vegetable image data, thus alleviating the problem of difficult labeling and insufficient samples of agricultural visual data [12].

At present, although the fruit and vegetable picking robot research has realized the initial transformation from “can pick” to “will pick” and “pick well”, it still faces many challenges and technical bottlenecks. First, many kinds of fruits and vegetables have different forms. The picking environment is complex and variable (such as changes in light, shading, overlap, and differences in the maturity of fruits and vegetables), which leads to the recognition algorithms based on deep learning in the generalization ability and the robustness of the recognition algorithms still having large room for improvement. Secondly, most of the existing picking robot systems are laboratory versions or have small-scale validation under specific conditions, and it is not easy to realize stable and efficient applications in large-scale agricultural scenarios. Some of the research focuses too much on algorithmic index (e.g., mAP, IoU) enhancement, while neglecting the evaluation of the overall performance of the whole picking system in real agricultural scenarios in terms of synergistic efficiency, energy consumption, cost, and other comprehensive performances. In addition, there is still a lack of systematic deep learning solutions for multi-sensor fusion, 3D vision reconstruction, and precise control of fruit grasping and picking movements. For example, data synchronization and feature fusion methods between multimodal data (RGB-D, LIDAR, IR, etc.) are not yet mature; robotic arm trajectory planning often relies on rules or predefined models, failing to combine visual information to achieve dynamic feedback control effectively, and most models have high requirements on computational resources and data volume during training, which is not conducive to deployment under edge computing conditions. Therefore, this paper provides a systematic review of the research on fruit and vegetable picking robots based on deep learning, comprehensively summarizes its latest progress in fruit detection, environmental adaptability, multi-sensor fusion, etc., analyzes the advantages and shortcomings of deep learning technology, and explores possible future research directions. This not only helps to provide an important reference for the research in related fields but also provides theoretical support and practical guidance for promoting the practical application of deep learning technology in fruit and vegetable picking robots.

In order to reflect the latest research results, this paper focuses on research results after 2020. More than 325 relevant scientific papers were retrieved from the Web of Science database using keywords such as deep learning, agriculture, and picking robots. Through further research and exclusion, 122 papers related to this topic were selected for in-depth study. Section 2 of the article provides an overview of deep learning technology; Section 3 analyzes the key technologies and applications of deep learning in picking robots in the literature; Section 4 focuses on optimization strategies and common problems related to deep learning; and Section 5 concludes the whole article with a summary and discussion of the challenges and future trends of deep learning technology.

## 2. Deep Learning Overview

Deep learning was proposed and developed during the research of artificial neural networks. Deep learning is one of the core technologies in the field of artificial intelligence, and its essence is an algorithmic framework based on multilayer neural networks, which can simulate the hierarchical abstraction and processing of data by the human brain. By constructing multilayer nonlinear computational units, deep learning can automatically extract features from large-scale data and perform pattern recognition, which is suitable for solving complex perception, classification, and prediction tasks [13,14]. In 2006, Hinton et al. [15] proposed an unsupervised greedy layer-by-layer training algorithm based on deep belief nets (DBNs). This proved that the deep neural network could be trained layer-by-layer after pre-training. After pre-training, it can achieve far better results than other methods in handwritten digit recognition tasks, which lays the foundation for the development of deep learning technology. Lecun et al. [16] proposed that convolutional neural networks, as an innovative multilayer learning architecture, are unique in effectively utilizing spatial positional relationship information. They thereby significantly reduce the number of network parameters and achieve a breakthrough improvement in backpropagation training efficiency. Currently, deep learning techniques mainly include convolutional neural networks (CNNs), recurrent neural networks (RNNs), generative adversarial networks (GANs), and deep reinforcement learning (DRL). Each of these algorithms has its own characteristics, with CNNs excelling in image processing and RNNs having an advantage in time-series data modeling; GANs are used for data generation and augmentation, and DRL excels in solving complex decision-making problems (Table 1). Listed below are several deep learning methods commonly used in picking robots.

### 2.1. Convolutional Neural Networks

**Figure 1:**
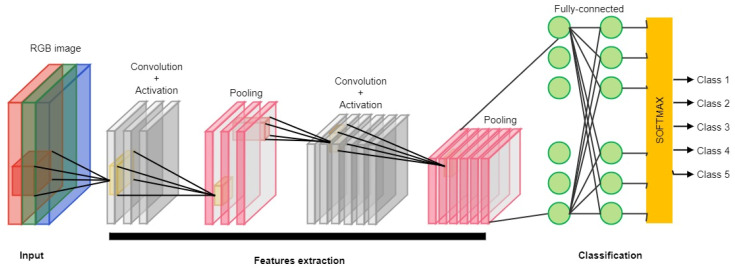
Convolutional neural network workflow diagram.

A convolutional neural network (CNN) is a type of neural network that specializes in processing grid topology data. The network has representation learning ability, which can realize hierarchical feature extraction and representation learning of images through operations such as local connectivity, weight sharing, and spatial dimensionality reduction. The convolutional neural network can learn and extract effective features automatically. It can fuse image information of different scales and extract multigranularity features through the multi-scale convolutional kernel and pooling window, which enhances its ability to understand complex scenes [17,18]. At the same time, the convolutional neural network has good generalization ability, using local connections and weight-sharing mechanisms, which greatly reduce the number of network parameters and model complexity. The detailed workflow of the convolutional neural network (CNN) is illustrated in Figure 1. However, the convolutional neural network relies on many labeled data. The computation required in training and inference is huge, resulting in high computational resources and storage space requirements. It is easily affected by noise, occlusion, and adversarial attacks in feature learning [19]. Despite some limitations, its powerful feature learning capability and excellent performance make it the model of choice in the field of image recognition. Future research directions include reducing the dependence on labeled data, improving the model’s interpretability and robustness, and reducing the computation and storage overhead to expand the application scope of CNNs further. In picking robots, CNNs are mainly used for visual perception, and most researchers have used CNNs to improve picking accuracy and efficiency around target detection and localization, fruit ripeness detection, picking path planning, and fruit quality grading [20,21,22,23]. Commonly used CNN architectures include AlexNet [24], VGGNet [25], GoogLeNet [26], and ResNet [27].

### 2.2. Object Detection

**Figure 2:**
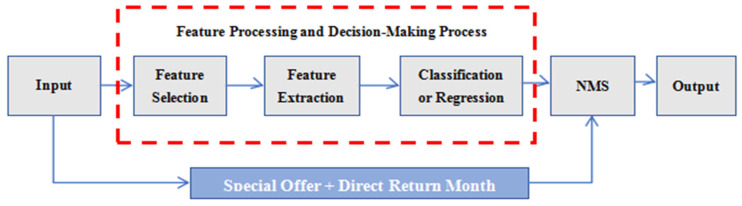
Object detection workflow diagram.

Target detection algorithms belong to an important branch in computer vision; by learning a large number of standard samples, they can accurately recognize and detect the category of the target object and where it is located and provide its bounding box edges [28]. The detailed workflow of the object detection is illustrated in Figure 2. Deep learning has spawned two mainstream algorithms in the field of object detection, including two-stage detection algorithms and one-stage detection algorithms [29]. The two-stage algorithm belongs to segment-to-segment target detection; firstly, it uses various convolutional neural networks as the backbone network and performs the feature extraction to generate the candidate region; then, secondly, it classifies and regresses the candidate region; the algorithm has high accuracy and is more robust to occlusion, deformation, etc., but the computational speed is slow. Mainstream two-stage algorithms mainly include RCNN [30], Fast R-CNN [31], Faster R-CNN [32], FPN [33], etc. The one-stage algorithm belongs to the end-to-end target detection. It uses CNN convolutional features to directly regress the object’s category probability and location coordinate values, which is less accurate than the two-stage algorithm, but it is simplified. The algorithm is less accurate than the two-stage algorithm, but it simplifies the process and is faster. Mainstream one-stage algorithms mainly include the YOLO series [34], SSD [35], etc. Target detection algorithms can quickly and accurately identify and localize mature targets in complex agricultural production environments for picking robots, providing support for picking decisions and path planning. It also plays an active role in recognizing obstacles and guiding the robot end effector to avoid obstacles. The commonly used target detection frameworks for general picking robots are SSD, YOLO, Faster R-CNN, etc. [36,37,38,39]. The generalization and robustness of the current target detection algorithms in terms of scene lighting problems, shooting angle, fruit shape, etc., need to be further optimized.

### 2.3. Deep Reinforcement Learning

**Figure 3:**
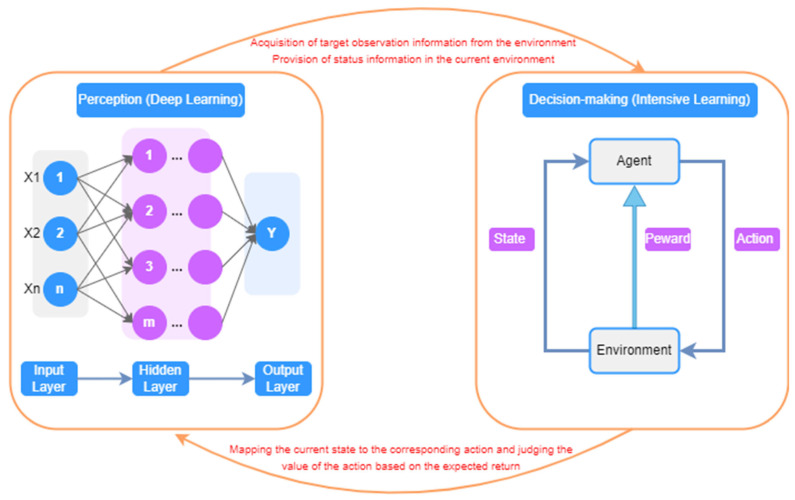
Deep reinforcement learning workflow diagram.

Deep reinforcement learning is a neural network-based machine learning method that combines deep learning and reinforcement learning to define the problem and optimization objective with reinforcement learning; it uses deep learning to solve the problems of state representation, policy representation, value function modeling, etc., and deep neural networks to approximate reinforcement learning value functions or policy functions, thus realizing end-to-end autonomous learning and decision making [40,41]. Deep reinforcement learning can adaptively extract helpful features in the face of complex, high-dimensional environments. With strong generalization ability, it can deal with the problem of continuous action space and be applied to intelligent robotics, natural language processing, image recognition, and other fields. The detailed workflow of the deep reinforcement learning is illustrated in Figure 3. The application in fruit and vegetable picking robots mainly plays an important research role in vision servo control, path planning strategy optimization, flexible end effector control, etc. Researchers have utilized deep reinforcement learning algorithms, such as DQN [42], DDPG [43], SAC [44], etc., combined with computer vision and multi-sensor fusion technology, to significantly improve the perception, decision making, and execution of picking robots’ ability to pick [45,46,47,48,49]. However, deep reinforcement learning algorithms require a large amount of environmental interaction data in practical applications; the training and learning process is slow, easily affected by hyperparameters, initialization, and other factors, and the model interpretability is poor, which limits its application effect. Future research needs to improve the efficiency and accuracy of the technique in methods such as data enhancement, multi-intelligence body collaboration, model interpretability, and combination with migration learning to better adapt to different tasks and environments.

### 2.4. Semantic Segmentation

**Figure 4:**
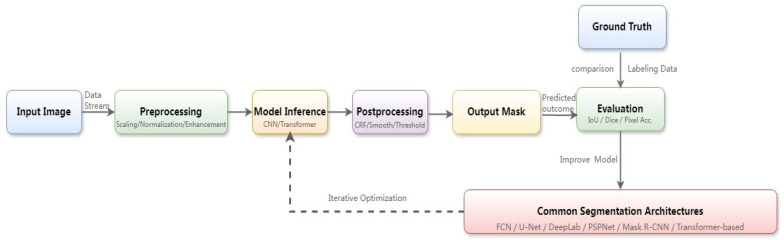
Semantic segmentation workflow diagram.

Semantic segmentation is an important application of deep learning in image processing, aiming to classify each pixel in an image, thereby determining the region or class it belongs to [50]. The detailed workflow of the semantic segmentation is illustrated in Figure 4. Commonly used semantic segmentation networks include FCN [51], SegNet [52], U-Net [53], DeepLab [54], etc., which usually adopt the network structure of encoder–decoder. The encoder is responsible for extracting the high-level semantic features of an image, and the decoder categorizes each pixel according to the feature mapping. Picking robots incorporating semantic segmentation can accurately identify and localize the features and attributes of target picks, which can be applied to target detection and localization, ripeness recognition, fruit and vegetable counting, obstacle recognition, and other aspects to improve the robot’s perceptual ability and picking accuracy [55,56,57,58]. However, semantic segmentation techniques have problems such as inaccurate segmentation accuracy and poor handling of fuzzy boundaries for targets with small areas or low frequency of occurrence, and enhancement strategies such as data augmentation and cost-sensitive learning are needed to improve the accuracy. In addition, since the end-to-end segmentation model relies on a large number of pixel-level sample data, the computational volume is large, and the real-time performance is poor, which makes it less efficient in practical applications. In the future, the robustness and efficiency of the technique can be improved by investigating lightweight models, small-sample learning training, and multimodal fusion.

## 3. Key Technology and Application of Deep Learning in Picking Robots

This section focuses on the key technologies and applications around deep learning in visual perception and target recognition, path planning and motion control, and intelligent control of end effectors for picking robots.

### 3.1. Visual Perception and Target Recognition

Visual perception and target recognition technology are the key to realizing intelligent picking robots. An efficient visual perception and target recognition system can help fruit and vegetable picking robots to quickly locate and recognize ripe fruits in complex and changing agricultural environments and provide reliable sensing information for subsequent path planning and end effector control, thus improving picking efficiency and success rate. This section focuses on the research progress and key technologies of deep learning techniques in fruit detection and classification and stem and leaf segmentation. Table 2 summarizes the latest research progress of deep learning in visual perception and object recognition.

#### 3.1.1. Fruit Detection and Classification

Fruit detection and classification aims to locate the spatial position of target fruits from images or video streams and further determine their attributes, such as variety category and maturity. Currently, mainstream deep learning target detection algorithms, such as Faster R-CNN, YOLO, SSD, etc., have been widely used in fruit detection and have been improved and optimized for agricultural scenarios, such as the introduction of multi-scale feature fusion, attention mechanism, etc., to improve the detection accuracy and robustness of the algorithm. In addition, some lightweight target detection models, such as YOLO-Tiny and MobileNet, are also used to build an efficient embedded visual perception system to meet the real-time demand of picking robots.

Researchers have improved and optimized the classical deep convolutional neural network target detection algorithms to meet the special characteristics of agricultural scenarios. Zhang et al. [59,60] proposed the HGCA-YOLO and YOLO5-Spear models to achieve high-precision real-time localization of white asparagus tips by designing modules to enhance the sensitivity of the target in complex backgrounds and a lightweight network structure. Xiao et al. [61] improved the YOLOv4-Tiny network by adopting multi-scale feature fusion, cavity convolution, and an anchor-free strategy to improve the detection capability of citrus small targets while taking into account the computational efficiency. Liu et al. [39] introduced the coordinate attention mechanism and BiFPN structure based on the YOLOv5s and optimized the detection head design to form the YOLOv5s-BC model, which significantly improved the apple detection accuracy. Chai et al. [62] were the first to integrate YOLOv7 with augmented reality technology for the purpose of strawberry ripeness detection in agricultural applications, achieving real-time detection at a rate of 18 ms per frame while maintaining high accuracy. Zeeshan et al. [23] used a general real-time dataset and convolutional neural network technology to accurately recognize orange targets. The model uses the RMSprop optimizer and ReLU activation function and adjusts the hyperparameters (e.g., batch size, number of layers, neuron number) to optimize the model performance, which achieves efficient detection and localization of orange fruits in dynamic environments.

The research has also explored multimodal perception fusion technologies to improve fruit detection and attribute analysis, enhancing the ability to perceive the internal attributes and spatial positioning of fruits. Ge et al. [21] combined the internal sensing system of a multi-view gripper with a lightweight convolutional neural network to realize a comprehensive assessment of strawberry ripening by fusing the front and back images of fruits to overcome the limitations of single-view perception. Liu et al. [38] proposed a multi-sensor fusion method based on ORB-SLAM3, YOLO-V5, and Lidar to achieve high-precision 3D localization of apple targets in dynamic environments through machine vision localization, image detection, and point cloud processing. These studies utilize the complementary characteristics of different sensors to obtain more comprehensive fruit information, expand the dimension of traditional visual perception, and effectively improve the precision control level of fruit picking robots.

To address the challenges of sample scarcity and scene variability in agricultural object detection, researchers have explored the application of small-sample learning and data augmentation techniques in this field. Lee et al. [63] evaluated the effect of RandAugment (RA) data augmentation in optimizing the crop detection model, and experiments have shown that applying an RA strategy to small fruit datasets, such as tomato and apple datasets, can effectively improve the model’s performance. Škrabánek et al. [64] combined data enhancement with the DenseNet network structure in a grape variety identification task to alleviate the small-sample problem while ensuring the generalization of the model. These works show that data augmentation and domain-adaptive small-sample learning strategies are effective ways to alleviate the problem of data scarcity in agriculture and can provide algorithmic support for the practical deployment of fruit picking robots.

The real-time performance of the visual perception module plays an important role in fruit detection. In order to meet the resource constraints of embedded devices, researchers have explored lightweight design methods for deep learning models. Ge et al. [21] proposed a MiniNet convolutional neural network, which reduces the inference time to 6.5 ms by optimizing the depth of the network and the size of the convolution kernel while guaranteeing the accuracy of maturity regression. Zhang et al. [60] designed the YOLO5-Spear model with a highly converged lightweight backbone network and optimized detection head structure to achieve 97.8% accuracy and real-time performance on the white asparagus tip detection task. Qi et al. [65] proposed a highly efficient detection architecture based on CSPDenseNet and a recursive feature pyramid for chamomile detection, achieving 47.23 FPS with 92.49% average accuracy to achieve a 47.23 FPS inference speed. The improved YOLO model of Brasilia et al. [66] can detect tree fruits in real time at a rate of more than 20 FPS. Kang et al. [67] achieved high-precision recognition and segmentation of apple fruits by introducing a lightweight Mobile-DasNet backbone in combination with the PointNet network, significantly reducing computational complexity while ensuring recognition accuracy. These works show that the lightweight network structure design and computational optimization techniques can effectively balance the accuracy and speed of the fruit detection task and provide an algorithmic foundation for the real-time sensing of fruit picking robots.

#### 3.1.2. Stem and Leaf Segmentation

In addition to the fruits themselves, the picking robot also needs to accurately recognize and localize the stems and leaves of the fruits and vegetables in order to achieve obstacle avoidance and accurate picking. Semantic segmentation plays a key role here, where each pixel in the image is labeled with a specific category. CNN-based models like FCN, U-Net, and DeepLab are widely adopted, and their performance has been enhanced using context modules and attention mechanisms. Research has also explored instance segmentation to handle overlapping leaves and branches, allowing more precise structural understanding.

Fujinaga et al. [68] realized multitasking for strawberry picking and stem pruning by combining DeepLabV3 with semantic segmentation models; the F1 scores for ripe and unripe fruit detection were 0.96 and 0.88 for ripe and unripe fruit detection, and the F1 scores were 0.93 and 0.86 for picking and pruning cutting point detection, respectively. Lemsalu et al. [69] developed a real-time, lightweight visual perception system based on YOLOv5 and realized strawberry and fruit stalk detection at 30 fps on a Jetson AGX Xavier edge device, with average accuracies of 91.5%, and Coll Ribes et al. [22] proposed an innovative method that combines monocular depth estimation with convolutional neural networks (CNNs) to achieve precise detection and localization of grape clusters and fruit stems in the complex environments of vineyards. This method was tested on two different vineyard scene datasets, and the results showed that its performance was superior to the current state-of-the-art methods. Li et al. [70] proposed a strawberry recognition method based on improved YOLOv7 and RGB-D sensing and introduced the integrated GhostConv module and CBAM attention mechanism module on the basis of YOLOv7, which optimized the detection performance of small targets, and the recognition accuracy was improved by 11–14.8%. Wang et al. [71] proposed a parallel network structure, the DualSeg model, which fused the advantages of the CNN and Transformer through the parallel double-branch structure and the features of the Transformer. Through the parallel dual-branch structure and feature fusion, it can segment grape bunches and fruit stalks more accurately in complex vineyard environments, and the IoU value of the grape stalk segmentation of this model is 72.1%, which is more than 3.9% higher than that of other models.

Researchers applied instance segmentation to the separation of overlapping leaves and branches to obtain finer structural information. Li et al. [72] proposed a multitask perceptual network, MTA-YOLACT, which added detection and instance segmentation branches to the pre-trained YOLACT model and used the shared ResNet-101 backbone network and loss function based on the uncertainty weights to achieve fruit bunch detection, fruit stalk main stem segmentation, and its subordination decision. Peng et al. [57] proposed a depth information-based region growing method, DRG, for segmenting overlapping grape bunches, which can effectively deal with the complex situation of multiple overlapping bunches and obtain a finer contour, with an average recall rate of 89.2% and an average precision rate of 87.5%.

Multitask learning approaches, which combine target detection, semantic segmentation, and pose estimation, have been explored to offer more comprehensive spatial and structural perception of fruits and vegetables. Jing et al. [73] proposed a three-dimensional localization method for kiwifruit with an end-to-end stereo matching network LaC-Gwc Net combined with a target detection model, YOLOv8m, and a better result was obtained for kiwifruit and its calyx. Good results were obtained with an average accuracy of 93.1% and a detection speed of 7.0 ms, which is especially important for sensing obstacle avoidance of small obstacles such as branches and wires. Kim et al. [74] introduced the PAF method in the field of human pose estimation into the pose estimation of a tomato fruit stalk system for the first time; the structural information of multiple tomatoes could be acquired from a single image simultaneously, and the average detection rates of the key points and segments were 92.9% and 92.9%, respectively. Miao et al. [75] proposed a visual perception method integrating YOLOv5, ROI extraction, and an adaptive weighting strategy for tomato picking, which combines the advantages of image processing and deep learning and realizes the estimation of tomato ripeness and accurate stalk localization under different light conditions, and the developed picking robot achieves the picking efficiency of one cluster in 9 s and the success rate of 95%. The developed picking robot achieves a picking efficiency of 9 s per cluster and a success rate of 95%.

**Table 2:** Comparison of different applications of deep learning in visual perception and object recognition.

Type	Ref.	Crop	Year	Task	Network Framework and Algorithms	F1 Score	Precision	mAP	Other Metric
Fruit detection and classification	[59]	White asparagus	2024	Object detection	HGCA-YOLO	92.4%	-	95.2%	
	[60]	White asparagus	2022	Object detection	YOLO5-Spear	96.8%	-	1	
	[61]	Citrus	2024	Object detection	YOLOv4-Tiny	-	93.5%	93.25%	Recall: 93.73%
	[62]	Strawberry	2023	Object detection	YOLOv7-Multi-Scale	92%	-	89%	
	[39]	Apple	2024	Object detection	YOLOv5s-BC	84.32%	99.8%	88.7%	
	[23]	Orange	2023	Object detection	CNN	96.5%	98%	-	Recall: 94.8%
	[21]	Strawberry	2023	Object detection	MiniNet CNN	-	-	-	MAE: 4.8%
	[38]	Apple	2023	target location	YOLO-V5, ORB-SLAM3	-	-	-	RMSE: 26 mm
	[63]	Tomato	2023	Object detection	YOLOv3, YOLOv8, EfficientDet	-	76.42%	93.73%	
	[64]	Grapevine	2023	Image segmentation	DenseNet	-	98.00 ± 0.13%	-	
	[65]	Tea Chrysanthemum	2021	Object detection	TC-YOLO	-	-	92.49%	
	[66]	Apple, pear	2019	Object detection	CNN	81%	95%	-	
	[67]	Apple	2020	Object detection	Mobile-DasNet	85.1%	-	86.3%	
Stem and leaf segmentation	[68]	Strawberries	2024	Cutting point detection	DeepLabV3+, ResNet-50	91%	-	-	MBF: 74.2%
	[69]	Strawberries	2022	Object detection	YOLOv5m	89.4%	89.0%	91.5%	Recall: 89.8%
	[22]	Table Grapes	2023	Object detection	Mask R-CNN	94.7%	95.6%	-	Recall: 93.8%
	[70]	Strawberries	2024	Object detection	YOLOv7	97.9%	99.0%	99.8%	Recall: 96.9%
	[72]	Cherry tomato	2023	Image segmentation	MTA-YOLACT, ResNet-101	95.4%	98.9%	45.3%	Recall: 92.1%
	[57]	Grape	2021	Image segmentation	DeepLabV3+	98.63%	87.5%	-	Recall: 89.2%
	[73]	Kiwifruit	2024	Object detection	LaC-Gwc Net, YOLOv8m	-	-	93.1%	
	[74]	Tomato	2023	Object detection	YOLOv5	-	-	91.7%	

### 3.2. Path Planning and Motion Control

The autonomous navigation of harvesting robots in orchards requires overcoming the challenges of environmental complexity and dynamic changes. Optimized path planning algorithms and smooth motion control are key to achieving this goal. By utilizing deep learning algorithms for optimization, harvesting robots can adjust their travel trajectory in real time in complex environments, reducing the risk of collisions. In addition, the optimized path can cover the entire work area, minimizing the possibility of omissions and repeated picking. Table 3 summarizes the latest research progress of deep learning in path planning and motion control.

#### 3.2.1. Deep Reinforcement Learning-Based Path Planning

In the field of picking robots, the core task of path planning is to comprehensively consider environmental information and task requirements and find a collision-free, optimal or suboptimal path for the robot from the starting point to the target point. The limitations of traditional path planning algorithms are becoming increasingly prominent in dynamic and complex environments. Algorithms such as Dijkstra’s often struggle to meet performance requirements in terms of real-time performance and robustness, resulting in severe constraints on robots’ adaptability and fault tolerance. In recent years, deep reinforcement learning (DRL) has shown great potential in the field of robot path planning by virtue of its powerful environment awareness and policy optimization capabilities. Through end-to-end learning, deep reinforcement learning can realize direct mapping from raw observation data to action output without designing complex rules manually and shows excellent adaptability and robustness in dynamic, uncertain environments. More and more scholars have started to apply deep reinforcement learning to the path planning task of fruit and vegetable picking robots.

Lin et al. [47] used a recurrent deep reinforcement learning (recurrent DDPG) algorithm, combined with a long short-term memory network (LSTM), to realize accurate trajectory prediction in complex scenarios by utilizing historical state information. The success rate of the collision-free path planning of recurrent DDPG was 90.90%, which was better than that of the traditional DDPG (19.32%) and the bi-directional RRT algorithm and two-way RRT algorithm (88.18%). Li et al. [48] proposed an intermittent stop point planning method based on the deep deterministic policy gradient (DDPG) to improve the picking efficiency and optimize the motion path of a dual-arm tomato picking robot in greenhouse environments. By generating a sequence of stop points, this method significantly reduces redundant movements of the robotic arms between multiple target points and increases the number of effective picks per unit time. Wang et al. [46] designed a path planning strategy that combines deep reinforcement learning. The research team first maps the position coordinates of kiwifruit to a two-dimensional plane, thereby obtaining visual information about the distribution of fruits in the orchard. Next, they transformed the path coverage problem in this scenario into a mathematical model of the traveling salesman problem (TSP). In order to optimize the traversal order of regions, they further proposed an improved DQN algorithm. This approach effectively shortens the coverage path and improves the efficiency of target point coverage. Overall, deep reinforcement learning empowers the picking robot with human-like autonomous decision making and adaptive ability. It also provides new ideas and methods for flexible path planning in an unstructured farmland environment. In the future, further improvement of the generalization ability and balancing the exploration and utilization of the strategy while ensuring high harvesting efficiency will be an urgent challenge to be tackled in this direction.

#### 3.2.2. Multimodal Environment Perception and Semantic Understanding

As the orchard scene is complex and dynamic, it is often difficult for a single sensor to perceive the surrounding environment comprehensively and accurately. Therefore, integrating the advantages of multiple heterogeneous sensors and extracting semantically rich multimodal feature representations is the key to the accurate perception and control of fruit and vegetable picking robots. The RGB camera directly simulates the human eye’s vision with high image clarity. Chen et al. [76] proposed a set of dynamic visual servo control frameworks and localized fruits in real time through the YOLOv4 target detection network, combined with the self-built map, to achieve stable and efficient autonomous positioning. Combined with the self-built map and autonomous localization, it achieves stable and efficient fruit approximation and continuous operation. At the same time, the depth camera can obtain the distance information of the target to solve the scale uncertainty problem. Wang et al. [77] designed a multitask convolutional network A3N, which skillfully integrates the pixel-level features of RGB images and depth maps, realizes the segmentation of fruit instances and the regression of the grasping position, and integrates them into the robotic perceptual decision-making system, which greatly improves the performance of the robot in complex scenarios. The success rate of grasping complex scenes is greatly improved. Lidar has a larger detection range and accuracy. Xiong et al. [78] constructed a point cloud processing pipeline on this basis through the combination of threshold segmentation, depth target detection, accurate analysis of the distribution of obstacles around the fruits, and the generation of the optimal fruit separation path for robotic arm motion planning. It can be seen that multimodal environment perception requires deep learning technology and traditional image vision to cooperate and synergize their advantages. However, it still faces many challenges, such as sparse samples, variable scenes, and limited computation. A lightweight and efficient multimodal feature learning framework and autonomous evolution of the online learning paradigm will be an important research direction in the future.

#### 3.2.3. Deep Learning-Based Machine Vision Localization

Accurate target localization is a prerequisite for picking robots to realize automated operation. However, the orchard environment has a complex background and large changes in light, and the fruits are often obscured and clustered, which makes it difficult to adapt to traditional methods such as color thresholding and contour detection. Deep learning, with its powerful feature extraction and classification capabilities, provides a new solution to the fruit localization problem. Kok et al. [79] proposed a new method for apple pose estimation. They first used the Mask R-CNN to realize the fine segmentation of fruits, then combined this with trunk detection to eliminate the background interference, and finally cleverly utilized the circular a priori of apples to construct a 2D to 3D pose mapping model; finally, they realized the spatial localization of apples under the monocular camera. For the occlusion problem in dense fruit clusters, Xiong et al. [78] combined deep target detection and point cloud postprocessing organically, returned to the 3D bounding box directly through end-to-end learning, and designed a point cloud clustering optimization strategy, which effectively improved the localization accuracy in dense scenes. Currently, deep learning is reshaping the traditional machine vision paradigm to explore end-to-end object localization and pose estimation methods. Although promising progress has been made, the non-rigid and fragile nature of the orchard environment puts forward higher requirements for localization accuracy and speed. Lightweight and efficient detection and tracking algorithms, multi-view joint optimization strategies, and position estimation models with good interpretability are the main directions for the future.

#### 3.2.4. Multitask-Oriented Robot Motion Planning

After grasping the global information of the orchard environment, it is necessary to further formulate the optimal picking motion strategy according to the constraints of fruit distribution and robotic arm structure. This is a complex combinatorial optimization problem involving multi-objective constraints in kinematics and dynamics. In recent years, deep learning has injected new vitality into traditional motion planning methods. Li et al. [80] proposed an integrated model (DRL-MPC-GNNs) based on deep reinforcement learning (DRL), model predictive control (MPC), and graph neural networks (GNNs), which solves the multi-robot system optimization problem by combining the powerful feature extraction capability of deep learning and the decision-making mechanism to solve the multitask motion planning problem of multi-robot systems in complex dynamic environments. Martini et al. [81] proposed a PIC 4 rl-gym modular framework combined with DRL implementation for training robots to perform multitask autonomous navigation planning in complex environments. Li et al. [82] proposed a new framework for collaborative multi-arm picking scheduling optimization, which first applies the improved Faster R-CNN detection network to realize multi-angle fruit recognition and localization and construct a global perceptual map accordingly. On this basis, a multi-arm task allocation algorithm based on deep reinforcement learning is designed. Through the end-to-end learning collaboration strategy, multiple robotic arms are guided to autonomously divide the work, minimize the overall operation time while avoiding collision and interference, reduce the repetitive movement and idling waiting time in the dense fruit area and narrow operation space, and improve the overall picking efficiency. In order to further improve the coverage picking efficiency, Wang et al. [46] divided the orchard into several regions and optimized the traversal order of each sub-region using the re-DQN network. By modeling it as a traveler’s problem that solves and leverages the high-dimensional approximation capability of deep reinforcement learning, the method shortens the global path length while also dramatically reducing the motion search complexity and improves the amount of picking per unit time by reducing unnecessary path backtracking and conflicts. With the improvement of the intelligence level of orchard operation, deep learning is reshaping the traditional robot motion control paradigm, injecting autonomous learning and optimization capabilities. However, ensuring the real-time aspects and robustness of planning while meeting various constraints, balancing local path optimization and global long-term gain, and organically integrating operations research optimization and deep learning are still challenges that need to be overcome in this field.

**Table 3:** Comparison of different applications of deep learning in path planning and motion control.

Ref.	Crop	Year	Task	Network Framework and Algorithms	F1 Score	mAP	Acc	Other Metric
[47]	Guava	2021	Path Planning	RNN, DDPG	-	-	-	Success Rate: 90.90%
[48]	Cherry Tomato	2024	Path Planning	YOLOv5, DDPG	95.7%	-	-	Detection speed: 16.5 FPS
[76]	Cherry Tomato	2024	Object detection	YOLOv4, ORB-SLAM3	-	-	-	Recall: 87.5%
[77]	Apple	2022	Object detection	YOLACT, Apple 3D Network	89%	-	-	IoU: 87.3%
[78]	Strawberry	2020	Object detection	Mask R-CNN	-	-	-	Success Rate: 65.1%
[79]	Apple	2024	Object detection	DaSNet-v2, HigherHRNet	-	89.11%	-	
[80]	-	2024	Path Planning	DRL-MPC-GNNs	92.91%	-	96.82%	Recall: 91.47%
[82]	Apple	2023	Path Planning	Multi-task DCNN	71.0%	67.3%	92.0%	Recall: 74.4%

### 3.3. Intelligent Control of End Effector

End effector intelligent control technology is the key for fruit and vegetable picking robots to realize accurate, efficient, and lossless picking operations. The current research on intelligent end effector control mainly focuses on perception and understanding, motion planning, force control, learning and adaptation, and task planning. Combining traditional control theory with artificial intelligence technology is the development direction of this field to continuously improve the autonomy, flexibility, and robustness of the robot end effector. This section focuses on grasping state classification and anomaly detection, optimal grasping attitude detection, obstacle avoidance path planning, and collision-free control, as well as target attribute sensing and force prediction. Table 4 summarizes the latest research progress of deep learning in intelligent control of the end effector.

#### 3.3.1. Target Attribute Extraction and Grip Force Prediction for Haptic Perception

Haptic perception plays an indispensable role in the process of fruit and vegetable picking. In recent years, researchers have begun to introduce deep learning into haptic perception to adaptively extract target attribute features from massive haptic data. Huang et al. [83] constructed a large-scale haptic dataset for agricultural applications. They proposed a deep neural network based on the Transformer, which extracts the key features of the haptic sequence in both spatial and temporal dimensions to realize target attribute perception and strength prediction in the process of grasping. Ma et al. [84] proposed a deep neural network based on the Transformer, which extracts the key features of haptic sequences from both spatial and temporal dimensions and realizes the target attributes and strength prediction in the grasping process. Ma et al. [84], Lin et al. [85], and Li et al. [86] investigated fruit hardness and grasping force from the perspectives of robotic opto-tactile sensors, active deformation of flexible fingers, the self-attentive capsule network, etc. Han et al. [87] explored the visual–tactile multimodal information fusion, further improving the grasping success rate and prediction accuracy. Zhang et al. [88] proposed a method targeting crops with inflorescence peduncles, wherein the Mask R-CNN model was integrated with a low-cost all-in-one gripper to enable simultaneous gripping and cutting of crop stems without contacting the fruit flesh. These studies utilize deep learning models to extract sparse, multi-scale, and cross-domain feature representations from tactile data, which, to a certain extent, overcome the difficulties of complex surface textures and large material differences between fruits and vegetables. However, the current research mainly focuses on the laboratory environment and how to expand it to the real orchard scene, which still faces many challenges; the future also needs to focus on overcoming the key challenges of active tactile strategy learning, tactile–motor control integration, fine classification of the grasping state, and more.

#### 3.3.2. Optimal Gripping Posture Detection by Fusing Visual Information

Determining the appropriate grasping angle is crucial for fruit and vegetable harvesting. Deep learning technology has recently been widely applied in grasping posture detection. By calculating the optimal grasping posture to guide the robotic arm’s picking action, the end effector’s positioning accuracy has been effectively improved, thereby increasing the success rate of harvesting. Ma et al. [89] improved the GG-CNN2 network; they proposed a method to predict the optimal grasping angle of kiwifruit, guided the manipulator to safely approach, grasp, and detach the kiwifruit, and combined it with the YOLO v4 target detection and achieved an 88.7% picking success rate. Sun et al. [90] proposed the FPVP, which is the most effective way to integrate visual information into grasping gesture detection. The FPENet multitask learning network proposed by Sun et al. [90] can simultaneously localize the position of the navel point and predict the 3D rotation vector of citrus. The CM-YOLOv5s-CSPVoVnet improved network designed by Wang et al. [91] can accurately localize the yellow peach gripping point in complex environments. Hewen Zhang et al. [92] used the YOLOv5s target detection algorithm combined with a depth camera to realize the transformation from a pixel coordinate system to a robotic arm-based coordinate system. Ni et al. [93] proposed a multimodal representation combining two visual modalities, the RGB image and depth map, and applied a two-way CNN to classify the grasping poses. These works mainly start from RGB images or RGB-D data and realize the accurate prediction of grasping parameters through end-to-end deep convolutional neural networks. Despite the good experimental results, there are more specialized detection models designed for different fruit and vegetable varieties and scenes, and there is an urgent need to develop unified frameworks and general algorithms and make breakthroughs in multimodal sensing information fusion and grasping gesture dynamic optimization.

**Table 4:** Comparison of different applications of deep learning in intelligent control of end effector.

Ref.	Crop	Year	Task	Network Framework and Algorithms	Precision	Acc	Recall	Other Metric
[83]	15 types of fruits	2023	Force Prediction	Transformer DNN	97.33%	97.33%	-	MAE: 0.216
[84]	Peach	2025	Force Prediction	CNN, CNN-LSTM	-	-	-	R^2^: 94.2%
[85]	Tomato, Nectarine	2023	Force Prediction	CNN-LSTM, ResNet	95.7%	97.8%	98.1%	IoU: 90.2%
[87]	Tomato	2024	Force Prediction	CNN, LSTM	95.7%	97.8%	98.1%	-
[89]	Kiwifruit	2022	Grab detection	GG-CNN2, YOLO v4	76.0%	-	-	Success rate: 88.7%
[91]	Yellow Peaches	2023	Object detection	CM-YOLOv5s-CSPVoVnet	87.8%	-	94.2%	-
[93]	-	2018	Grab detection	Two-stream CNNs	93.4%	-	77.2%	-

#### 3.3.3. Collision-Free Control of Robotic Arm Attitude

Realizing obstacle avoidance grasping in complex environments is another challenge for fruit and vegetable picking. In recent years, reinforcement learning has provided new ideas for solving this challenge. Li et al. [45] proposed a deep reinforcement learning-based collision-free control method of robotic arm attitude for tomato picking in complex environments with sequential obstacle avoidance grasping problems. The method combines the optimal operating attitude plane and the HER-SAC strategy. It dynamically generates collision-free robotic arm motion sequences through the improvement of attitude constraints, heuristics, and dynamic gain modules and achieves a picking success rate of 85.5% in greenhouse experiments, and analyzed from the perspective of the picking experiments and efficiency, the method significantly reduces the time delay due to the collision detection and replanning. Tabakis et al. [94], Lin et al. [95], and Choi et al. [96] proposed a deep reinforcement learning-based path planning method for a robotic arm by training in a simulated environment; the robotic arm was able to find the optimal path in a complex environment while avoiding collisions. This work shows that deep reinforcement learning can adaptively generate obstacle avoidance motion sequences through end-to-end policy learning, providing a new solution for fruit and vegetable picking in complex environments. In the future, it is necessary to improve the algorithm’s sampling efficiency and generalization ability further and make breakthroughs in multi-intelligent body cooperative obstacle avoidance, dynamic environment perception, and prediction.

#### 3.3.4. Deep Learning-Based Collaborative Energy Efficiency Optimization for Multiple Robots

From the perspective of energy efficiency optimization, deep learning techniques show great potential in the cooperative control of the end effector of multi-picking robots. Aiming at the problem of collaborative energy efficiency optimization of the end effector in a multi-robot system, Zhao et al. [97] proposed a deep reinforcement learning framework for multi-robot collaboration based on proximal policy optimization (PPO); they systematically analyzed the effects of different types, magnitudes, and distributions of perturbations on the learning efficiency and laid a foundation for constructing a highly energy-efficient end effector collaborative system adapted to the uncertainties of real orchard environments. Ao et al. [98] proposed a dual-stream attention actor-critic (DSAAC) algorithm based on multi-intelligence deep reinforcement learning, which enables the system to automatically learn the optimal strategies in complex environments, strike a balance between dynamic allocation of communication loads and trajectory planning, and effectively solve the energy-constrained problem in emergency communication scenarios. Wu and Wu et al. [99] proposed a communication embedded deep Q-network (CE-DQN) for distributed multi-robot control, which enables robots to make autonomous decisions on when to share local information and effectively solves the energy- and time-consumption problems in multi-robot systems. Xiao et al. [100] proposed a collaborative inference framework for mobile edge computing based on multi-intelligent deep reinforcement learning (DMECI), which enables mobile devices to self-adaptively select partition points of deep learning models and collaborative edge servers, combined with a learning experience exchange mechanism to achieve knowledge sharing among neighboring devices, enabling intelligent collaborative computing resource allocation and energy efficiency optimization without relying on inference performance models. These deep learning-based approaches improve the energy efficiency of multi-end effector systems and enhance the adaptability of the end effector to complex fruit and vegetable picking environments. Future research can explore more advanced deep learning architectures, such as deep spatio-temporal graph networks and multimodal Transformers, to enhance the end effector’s intelligence and energy efficiency in fruit and vegetable picking systems.

### 3.4. Summary

In summary, deep learning technology shows great potential and practical value in the three modules of visual recognition, path planning, and end effector control of fruit and vegetable picking robots. In visual recognition, it is recommended to adopt advanced convolutional neural network models such as YOLO and Faster R-CNN combined with attention mechanism and image enhancement technology to achieve high-precision recognition and ripeness determination of different fruit and vegetable categories in complex environments and, at the same time, introduce migration learning and multimodal fusion strategies (e.g., RGB-D images and LiDAR point clouds) to enhance the model’s generalization ability and 3D spatial localization accuracy. We also adopt lightweight models such as MobileNet or EfficientNet to balance model performance and deployment efficiency. Regarding path planning and motion control, traditional planning methods are difficult to adapt to the dynamics of unstructured orchard environments. It is recommended to introduce deep reinforcement learning (e.g., PPO, DDPG, etc.) to achieve self-learning and self-adaptive path optimization and to cooperate with simulation platforms for model training and imitation learning to enhance the generalization ability of the strategy and to incorporate SLAM technology and graph neural networks for environment mapping and localization to further improve the navigation accuracy and dynamic obstacle avoidance ability. In terms of end effector control, due to the wide variety of fruits and vegetables and large structural differences, it is necessary to have flexible and fine control capabilities. It is recommended to train a multimodal perception network by integrating visual and tactile sensors and introduce deep reinforcement learning to realize a dynamic force–control strategy based on feedback, so that the robot can adaptively adjust the grasping posture and strength according to the shape and growth state of the fruits and thus effectively reduce the damage rate of the fruits.

In the future, with the development of algorithms and hardware technology, the introduction of deep learning will significantly promote the fruit and vegetable picking robot from “usable” to “efficient and reliable”, providing strong technical support for modern intelligent agriculture. Table 5 compares and analyzes the key technologies of deep learning-based harvesting robots.

## 4. Training and Optimization of Deep Learning Models in Picking Robots

### 4.1. Dataset Construction and Labeling Methods

A high-quality dataset is a prerequisite for training high-performance deep learning models. Constructing high-quality datasets faces many challenges when studying fruit and vegetable picking robots. First of all, there are many kinds of fruits and vegetables, and different kinds and varieties of fruits and vegetables have significant differences in shape, size, color, etc.; so, it is necessary to collect data samples that cover a variety of variations. Second, the growing environment of fruits and vegetables is complex and variable; light, shade, background, and other factors will affect the data quality. In addition, the picking robot needs to judge the ripeness and integrity of fruits and vegetables, which requires the dataset to contain samples of fruits and vegetables at different growth stages and with different degrees of integrity.

To address the above challenges, researchers have proposed various dataset construction methods. Zhang et al. [60] proposed a multi-scale combined slicing and resampling-based image transformation enhancement method for selective harvesting of white asparagus and constructed datasets in rainy and non-rainy day scenarios to expand the training test samples. Wang et al. [75] utilized a depth camera and an RGB-D camera for the training tests on a rainy day and a non-rainy day scenario. RGB-D cameras, 768 RGB-D images and 1132 color images were collected at different times and distances in an apple orchard for training the model; Hwang et al. [101] investigated the creation of the HWANGMOD dataset and generated a large number of 3D citrus images in virtual and real environments through Blender and BlenderProc model data to provide support for model training. Zhang et al. [102] collected tomato image data at five ripening stages to create a dataset through image annotation and grading. Geometric transformations and added noise were used for data enhancement, and the t-SNE method was used to verify the data distribution and clean up the unqualified samples; Qi et al. [103] proposed the Tea Chrysanthemum generative adversarial network (TC-GAN) for generating high-resolution images of Tea Chrysanthemums in a complex and unstructured environment (512 × 512); Zeeshan et al. [23] created a diverse dataset containing actual conditions such as different lighting, shadows, and blade overlaps, enhancing the algorithm’s adaptability and robustness in real-world scenarios. Sa et al. [104] integrated a public dataset with a self-constructed sweet pepper dataset and employed a generative adversarial network (GAN) to synthesize near-infrared (NIR) images from RGB inputs. By incorporating these synthesized images into a four-channel fruit detection framework based on YOLOv5, they provided highly adaptable training data for multimodal detection tasks. Table 6 lists the datasets and their processing methods used in some articles.

Regarding data annotation, the mainstream methods include bounding box annotation and semantic segmentation annotation. Bounding box annotation is to frame the location of target fruits and vegetables in the image and label their categories, such as apples, oranges, etc. The semantic segmentation annotation method achieves object recognition by labeling the category of each image point. Specifically, researchers annotate the collected raw data pixel by pixel when processing crop images to distinguish target objects such as fruits and vegetables from the background. Compared with bounding box annotation, semantic segmentation annotation can provide finer target contour information, but the annotation cost is also higher. Dai et al. [105] captured 960 images of tobacco leaves, increased the image data by rotating, scaling, and luminance change hoods, and labeled four different maturity levels of tobacco leaves. Kim et al. [106] proposed an automated data generation method to generate tomato datasets containing different maturity levels and bit positions through a 3D simulator, which avoids the cost of manual labeling and significantly improves the efficiency of training data generation. Wang et al. [107] captured 1260 images of Shine Muscat and Megapolis grapes. They used the professional annotation software Labelme 5.0.1 to label both the grapes and their stems, aiming to extract more comprehensive grape features.

### 4.2. Model Training Strategy

In terms of training strategy, fine-tuning is an effective means of improving the performance of fruit and vegetable detection. Selecting an ImageNet pre-trained backbone network (e.g., ResNet) and fine-tuning the self-constructed fruit and vegetable dataset can be significantly better than training from scratch. Secondly, some training techniques, such as data-balanced sampling, multi-scale training, learning rate warmup, etc., also help to further improve the performance and accelerate the convergence. In the training process, the selection of hyperparameters, such as learning rate, batch size, regularization parameter, and so on, is crucial. In order to optimize the hyperparameters, some researchers adjust the batch size, the number of layers, the number of neurons, etc., to optimize the model performance and search for the optimal combination of hyperparameters [23]. Sa et al. [10] adopted a two-phase training strategy: first, pre-training the backbone network on ImageNet to learn the general-purpose features, then fine-tuning it on a specific fruit dataset to extract the fine-grained features. In addition, hard example mining and online bootstrapping are also introduced for hard sample mining and training sample equalization to improve the detection accuracy. Suchet Bargoti and James Underwood [108] designed an iterative training strategy to augment the training data with the a priori knowledge of fruits and to iteratively update the detector.

When the self-constructed dataset is limited, a migration learning strategy can be considered to migrate knowledge from related domains and reduce labeling dependency. With models pre-trained on large-scale datasets (e.g., ImageNet), generic visual feature representations can be learned. In target tasks (e.g., fruit and vegetable detection), the feature extraction capability of the pre-trained models can be utilized to fine-tune them on smaller datasets for better performance. For example, Károly et al. [109] introduced a novel “Filling the Reality Gap” (FTRG) approach using automatically generated synthetic data to improve the fine-tuning phase of transfer learning in a target segmentation task, and Nguyen et al. [110] proposed a forward progressive learning (FPL) approach to achieve reliable transfer learning in robotics applications.

### 4.3. Model Optimization and Deployment

Although deep learning models have performed well in fruit and vegetable picking tasks, they still face challenges such as high model complexity, slow inference speed, and high resource consumption in practical deployment. To solve these problems, researchers have proposed several methods for optimizing models, including model lightweight, edge computing, and testing and evaluation using multiple models.

Model lightweight is a commonly used optimization method to reduce the storage and computation overhead of the model by reducing the number of parameters and the computation of the model. In the fruit and vegetable detection task, MobileNet [36,111] and other lightweight network structures are widely used; through the depth separable convolution and other techniques, the number of parameters of the model and the computational effort is greatly reduced while maintaining high precision. By improving the main network of CSPDenseNet, embedding the feature fusion module, and optimizing the multi-scale detection head, the number of parameters and the computational cost of the model are significantly reduced while guaranteeing the detection accuracy, and a lightweight and highly efficient Camellia sinensis detection network is finally realized.

In terms of model deployment, edge computing is a promising deployment method. Compared with cloud deployment, edge computing can deploy the model on edge devices close to the user, such as mobile devices, embedded devices, etc., which reduces the delay and cost of data transmission and improves the real-time aspects and privacy of the model [112]. Researchers have explored various edge computing schemes in fruit and vegetable picking robots. For example, Xiao et al. [100] tested real-time inference by putting the deep learning model on an NVIDIA Jetson TX2 edge device(made in NVIDIA Corporation, Santa Clara, CA, USA), with an inference time of about 0.1 s per image of 512 × 512 resolution, laying the foundation for realizing rapid deployment and updating of the model on robots in the future. Zhang et al. [113] proposed using a deep learning model using fine-grained computational partitioning to distribute the computational tasks of deep neural networks to edge devices and the cloud to optimize the end-to-end latency.

In addition to model optimization and deployment, model testing and evaluation are crucial to ensure model performance and reliability. In the fruit and vegetable picking task, researchers have used a variety of evaluation metrics and testing methods. For example, Wang et al. [114] proposed an evaluation framework based on a confusion matrix, which comprehensively evaluates the model’s performance by analyzing its performance in different categories and scenarios. In contrast, Tituaña et al. [115] proposed a testing method based on adversarial attacks, which evaluates the robustness and security of the model by generating adversarial samples.

### 4.4. Analysis of Model Adaptability and Scalability

Although deep learning technology has significantly promoted the intelligent development of fruit and vegetable picking robots, their adaptability and scalability in complex and changing orchard environments still face challenges. The spatial and temporal dynamics and heterogeneity of the orchard (e.g., light changes, crop growth differences, shading, and interference) significantly affect the robot’s recognition and operation performance, and it has become a research priority to improve the system’s robustness and generalization ability under multi-environmental conditions. First, the spatial distribution of crops significantly affects the visibility and occlusion of fruits, and deep learning can realize accurate detection of target fruits in complex backgrounds by convolutional neural networks (CNNs). For example, RGB images combined with Mask R-CNN networks were used for apples hanging high in the sky to achieve stable recognition under different lighting conditions [116]. For strawberries growing close to the ground and often occluded by foliage, it is necessary to introduce an attention mechanism and an instance segmentation network to improve the robustness and accuracy of occlusion. The SGSNet model developed by Wang et al. combines a lightweight network structure and an attention mechanism to effectively improve the visibility and accuracy of strawberries growing in the ground. The SGSNet model developed by Wang et al. combines a lightweight network structure and an attention mechanism, which effectively improves the detection accuracy of strawberry growth stages [117]. Second, the ripening process of different crops exhibits varied visual feature changes, such as color, texture, and fruit surface state. Deep learning effectively supports fine-grained discrimination of strawberry and grape ripening stages by modeling fruit ripening dynamics through time-series modeling (e.g., LSTM or Transformer) or combining hyperspectral images for non-invasive analysis. For example, Su et al. realized real-time estimation of strawberry ripeness using hyperspectral imaging combined with a deep learning model with a classification accuracy of more than 84% [118]. At the execution level, deep neural networks can be used for robotic gripping point prediction and path planning, combined with visual feedback to achieve adaptive clamping control for different fruit shapes (e.g., spherical for apples, bunches of grapes, and conical for strawberries) to minimize mechanical damage. In order to improve the generalization ability of the robot under different geographical and seasonal conditions, migration learning and incremental learning mechanisms are also widely used, so that its perceptual model can be quickly adapted to new crop varieties or new environmental conditions.

For cost analysis, although picking robots show great potential in enhancing agricultural automation, their commercialization faces significant cost challenges, especially for small and medium-sized farms, where higher initial investment and ongoing operation and maintenance costs are the main barriers to their popularity. Therefore, optimizing the overall cost structure through deep learning technology is particularly important. At the hardware level, the initial acquisition cost of the system can be reduced by choosing cost-effective sensors (e.g., consumer-grade high-resolution cameras and low-cost LIDAR) and adopting multiple alternative technologies. At the software level, optimizing deep learning models is one of the core strategies to reduce the computational resource requirements and operating costs. Current mainstream lightweight neural network architectures, such as MobileNet and EfficientNet, have been widely used in embedded platforms and resource-constrained smart devices. MobileNet significantly reduces the model parameters and computation volume by introducing deep separable convolution to efficiently support mobile vision tasks [119]. EfficientNet further proposes a compound scaling strategy to achieve a balanced scaling between model depth, width, and input resolution, which achieves a better accuracy–efficiency balance across multiple vision tasks [120]. This type of network architecture is suitable for deployment in agricultural picking robots, helping to reduce their dependence on high-power GPUs or the cloud, thus saving operational energy and costs. In addition, the maintenance cost of picking robots can be controlled by introducing AI-based fault prediction and diagnosis mechanisms to monitor the device operational status and predict potential failures in real time, thus extending the device lifecycle and reducing unplanned downtime.

## 5. Challenges and Future Trends

The rapid development of deep learning technology has injected new vitality into the research of fruit and vegetable picking robots; however, its application to real-world scenarios still faces many technical challenges. The complex and variable agricultural environment puts forward strict requirements on the robustness and generalization ability of sensing algorithms; the differences in heterogeneous sensor data from multiple sources exacerbate the difficulty of multimodal information fusion; the scarcity of agricultural scene samples contradicts the demand for deep learning data; and the key technologies such as semantic comprehension of the agricultural context and human–robot collaboration are not yet mature. For example, in the multi-vehicle cooperative fruit and vegetable picking task, some studies try to introduce a comprehensive multi-population genetic algorithm for task allocation, but in the real drift scenario, the lack of environment modeling and multi-objective trade-off imbalance results in the algorithm frequently falling into the local optimum in the high occlusion and fruit and vegetable dense area, which makes it difficult to achieve efficient operation [121]. In the face of the above challenges, the future development of fruit and vegetable picking robots needs to seek transformative breakthroughs in perception, integration, learning, planning, and interaction. By exploring the self-supervised learning paradigm to enhance the perception robustness, optimizing the multi-sensor cooperative perception framework to improve the quality of information fusion, exploring the new frontier of small-sample learning to ease the data demand, developing data-driven planning methods for dynamic environments to enhance the decision-making intelligence, and constructing an intelligent interaction mode in the context of agriculture to promote human–machine collaboration, we can encourage the fruit and vegetable picking robots to make leapfrog progress for the transformation of intelligent agriculture and empower the future development of fruit and vegetable picking robots.

### 5.1. Robust Perception Algorithms for Complex Environments

The research focus of future fruit and vegetable picking robot perception algorithms will be to improve the robustness and adaptability in complex and dynamic farmland environments. On the one hand, self-supervised learning, continuous learning, and other paradigms should be explored in depth, and unstructured environmental data should be used to learn more generalized visual feature expressions to reduce dependence on large-scale labeled data. On the other hand, combining the knowledge of the agricultural field to build a finer a priori model of the scene guides the deep learning algorithm to adapt to different farmland environments quickly. At the same time, the active learning strategy is used to optimize the sampling process dynamically, focus on complex cases for key learning, and continuously improve the environmental adaptation ability of the perception algorithm. For example, in the case of perceptual failure under hazy weather in an orchard, it was found that introducing an active sampling method based on a confidence regression mechanism effectively reduced perceptual errors [49].

### 5.2. Deep Multimodal Information Fusion Framework

Efficient fusion of visual, depth, near-infrared, and other multi-source sensory information is the key to improving the ability to understand fruit and vegetable picking robots. Future research should focus on constructing a unified deep multimodal fusion framework, realizing the entire interaction of multimodal data in the feature learning stage and capturing the intrinsic semantic association between different modalities. An attention mechanism should be introduced to dynamically adjust the importance of various modalities and suppress redundant information. At the decision-making level, it is appropriate to use probabilistic graphical modeling and other methods for global inference of multimodal information to obtain semantically consistent and robust fusion representations. Global reasoning, such as Bayesian graphical models, can be used at the decision-making level. For example, in an existing citrus picking experiment, a multimodal fusion approach based on graph neural networks reduced the false recognition rate by 18% in the occluded region compared to the traditional weighted average strategy, demonstrating its advantages in coping with uncertainty in agricultural environments [122]. Considering the specificity of agricultural scenarios, the framework should be fault-tolerant to incomplete and noisy inputs to ensure the continuity and stability of the sensing process.

### 5.3. New Paradigm for Small-Sample Learning in Agriculture

The development of a new paradigm of small-sample learning for agricultural applications is a meaningful way to break through the bottleneck of rapid deployment of fruit and vegetable picking robots. Meta-learning is a promising idea that acquires task-independent prior knowledge through a two-layer optimization process and then quickly adapts to new tasks according to the support set. Combining meta-learning with agricultural scenarios, constructing a priori libraries of farmland environments, and using a small number of target fruit and vegetable samples to fine-tune and form an exclusive model of picking are expected to improve the generalization of perception algorithms greatly. Knowledge distillation is another direction worth exploring. It can condense the feature representations learned from different fruit and vegetable picking models into a shared teacher network and then distil them into a small student network to improve reasoning efficiency while alleviating the data demand.

### 5.4. Data-Driven Autonomous Operation Planning

New data-driven job planning methods are urgently needed for the farmland’s complex and changing environment. Deep reinforcement learning (DRL) shows great potential for learning optimal harvesting strategies. By designing a reasonable reward function and embedding safety constraints, the intelligent body can autonomously learn obstacle avoidance path planning and picking action sequence optimization skills in the simulation environment and then transfer them to the actual robot. At the same time, the modeling capability should be further strengthened, and the simulation environment should be continuously optimized by using accurate data to narrow the gap between reality and simulation. At present, we still face the problem of the “simulation-reality migration gap”. Taking the multi-vehicle task assignment as an example, in the drifting orchard environment, there is “right-of-way conflict” and “path duplication” when using the reinforcement learning strategy to cooperate with multi-vehicles, resulting in local congestion; so, we should further strengthen the modeling ability, use real data to continuously optimize the simulation environment, and reduce the gap between reality and simulation. Therefore, the modeling capability should be further strengthened, and the simulation environment should be continuously optimized with the measured data to narrow the gap between reality and simulation. At the execution level, the planning system should be able to process dynamic feedback in real time, dynamically adjust the planning path according to the growth state of fruits and vegetables, the location of obstacles, etc., and ensure the robustness of autonomous operation with intelligent adaptability.

### 5.5. New Mode of Human–Computer Interaction in Intelligent Agriculture

Accompanied by the rapid development of natural language processing, knowledge mapping and other artificial intelligence technologies, fruit and vegetable picking robot human–machine interaction is expected to move towards a more intelligent and natural new stage. In the future, we should focus on the specific semantic understanding of agriculture, integrate the knowledge base of the agricultural field with the robot execution model, and realize the accurate recognition and mapping of agricultural terms. In terms of visualization, we should explore introducing knowledge mapping, virtual reality, and other technologies into the design of human–machine interfaces to provide users with a more intuitive image of multi-dimensional information presentation. At the level of human–robot collaboration and research on the active interaction paradigm, the robot actively seeks human help and dynamically adjusts the original strategy according to the feedback.

## Figures and Tables

**Table 1:** Summary of key deep learning methods commonly used in picking robots.

Method Category	Typical Networks	Mechanism	Advantage	Limitation
Convolutional neural network	LeNet, AlexNet VGGNet, ResNet	Hierarchical feature extraction and representation learning of images through local connectivity, weight sharing, and spatial dimensionality reduction.	Automatically learns rich features from a large amount of image data and performs well in tasks such as fruit detection and ripeness determination.	Dependent on a large amount of labeled data; the amount of computation required in training and inference is huge, leading to high requirements for computational resources and storage space; it is susceptible to noise, occlusion, and adversarial attacks in feature learning.
Target detection	YOLO Series, SSD RCNN, FastR-CNN	Through end-to-end feature learning, candidate region generation, classification, and regression, the mapping from images to target bounding boxes and categories is realized.	Quickly and accurately identifies and locates mature targets in complex agricultural production environments to support picking decisions and path planning	Generalization and robustness to be further optimized with respect to scene lighting issues, shooting angle, fruit shape, etc.
Deep reinforcement learning	DQN, DDPG, SAC	Approximating the value function or policy function of reinforcement learning through deep neural networks, thus realizing end-to-end autonomous learning and decision making.	Adaptive extraction of useful features in the face of complex, high-dimensional environments. Strong generalization ability, able to deal with problems such as continuous action space.	Requires a large amount of environmental interaction data, slower training and learning process, easily affected by hyperparameters, initialization and other factors, poor model interpretability, limiting its application effect.
Semantic segmentation	FCN, SegNet, U-Net, DeepLab	Each pixel within the image is systematically classified to ascertain the specific region or category to which it belongs.	It is capable of achieving extremely high target recognition and classification accuracy at the pixel level, accurately distinguishing between different target classes.	Due to the reliance on extensive pixel-level sample data, the computational requirements are substantial, leading to suboptimal real-time performance and reduced efficiency in practical applications. For targets with small areas or low frequencies, issues such as inaccurate segmentation and inadequate handling of fuzzy boundaries arise.

**Table 5:** Summary of comparative analysis of key technologies based on deep learning in harvesting robots.

Technical Category	Technology Type	Main Advantages	Main Disadvantages	Representative Technologies (Frameworks/Methodologies)
Visual perception and target recognition	CNN-based Classification/Detection	Strong feature representation, adaptable to various fruits	Large model size, slow inference, difficult to deploy	ResNet, EfficientNet
	YOLO Series Object Detection	Fast detection, suitable for real-time systems	Poor accuracy for small/occluded targets	YOLOv5, YOLOv7, YOLO-NAS
	Attention Mechanisms (Spatial/Channel)	Focuses on key areas, improves recognition in cluttered scenes	Increased complexity, harder to train	CBAM, SE, Transformer Attention
	Multimodal Fusion (RGB + D)	Strong spatial understanding, better in occluded scenarios	Complex calibration, high hardware cost	DenseFusion, PointNet, RGB-DNet
	Self-Supervised/Few-Shot Learning	Less dependence on annotations, fast adaptation	Weak generalization, unstable training	SimCLR, Meta-RCNN, FSL-YOLO
Path planning and motion control	Deep Reinforcement Learning (DRL)	Learns adaptive paths and obstacle avoidance	Slow convergence, needs large-scale simulation	PPO, DDPG, SAC, TD3
	GNN + Reinforcement Learning	Learns orchard topology, enables intelligent decision making	Complex architecture, hard to train	GNN-RL, GraphNav, GAT-RL
	Imitation Learning	Efficient training from expert demonstrations	Lacks exploration, cannot self-correct errors	Behavior Cloning, DAgger
	Deep Visual Navigation	No map needed, end-to-end instruction prediction	Low fault tolerance, image quality dependent	Deep Visual Planner, VNNet
	Learning-Based SLAM	Supports dynamic environment mapping and navigation	Complex fusion, hard to deploy	DL-SLAM, Neural SLAM, DROID-SLAM
Intelligent control of end effector	Deep Learning-Based Force Control	Adjusts gripping force automatically, reduces damage	Requires accurate sensors and data	DeepForce, GripNet, TactileCNN
	Multimodal Fusion (Vision + Tactile + Force)	High adaptability, robust grasping	Complex system integration, real-time challenges	Visuo-Tactile Fusion Net, GelSightNet
	Reinforcement Learning for Grasping	Learns grasping strategies for different shapes	High training cost, low sample efficiency	QT-Opt, VPG, RL-GraspNet
	Generative Models for Grasping (VAE/GAN)	Predicts grasp strategies under incomplete observations	Unstable inference, hard to train	GraspGAN, VAE-Grasp
	Graph-Based Grasping Strategy (GNN)	Suitable for fruit clusters, captures structural features	Complex modeling, limited real-world application	GGCNN, GAT-Grasp

**Table 6:** The datasets and their processing methods used in some articles.

Dataset	Data Volume	Data Acquisition Methods	Data Processing Methods
White asparagus dataset	6498 images (4248 non-rain enhanced + 2250 rain enhanced)	Image acquisition was performed using an industrial camera (Basler acA2500-14gc, made in Basler AG, Ahrensburg, Germany) with an image resolution of 2540 × 1920 pixels.	Multi-scale combined images, resampling-based image transformation
Apple orchards dataset	768 RGB-D images, 1132 color images	Using the Intel RealSense D435 RGB-D camera (made in Intel Corporation, Santa Clara, CA, USA)	Spatial conversion, color distortion, point cloud processing
Citrus Orchard Dataset	10,000 images	Using the Intel RealSense D435 RGB-D Camera (made in Intel Corporation, Santa Clara, CA, USA)	Using BlenderProc in conjunction with Blender to generate virtual datasets
Tomato Dataset	200 images	Manual shooting	Geometric transformations, random noise
Tea Chrysanthemum Dataset	26,432 images	Using an Apple X phone (made in Apple Inc., Cupertino, CA, USA) with an image resolution of 1080 × 1920	TC-GAN
Orange Dataset	2000 images	Using the Azure Kinect RGB-D camera (made in Microsoft Corporation, Washington, DC, USA) with a resolution of 1920 × 1080	Flip, rotate, and change lighting on images
Bell Pepper Dataset	1615 pairs of RGB + NIR images	Using the multispectral camera acquisition	Synthesis of NIR image from RGB image using GAN

## Data Availability

The data presented in this study are available on request from the corresponding author.
